# Rabies Situation in Cambodia

**DOI:** 10.1371/journal.pntd.0000511

**Published:** 2009-09-08

**Authors:** Sowath Ly, Philippe Buchy, Nay Yim Heng, Sivuth Ong, Nareth Chhor, Hervé Bourhy, Sirenda Vong

**Affiliations:** 1 Epidemiology and Public Health Unit, Institut Pasteur in Cambodia, Phnom Penh, Cambodia; 2 Virology Unit, Institut Pasteur in Cambodia, Phnom Penh, Cambodia; 3 Calmette Hospital, Phnom Penh, Cambodia; 4 Lyssavirus Unit, UPRE Lyssavirus Dynamics and Host Adaptation, WHO Collaborating Center for Reference and Research on Rabies, Institut Pasteur, Paris, France; University of Oklahoma Health Sciences Center, United States of America

## Abstract

**Background:**

Rabies, a fatal but preventable zoonosis, is a major public health problem in developing countries. In Cambodia the disease burden is largely underestimated because patients with encephalitis following dog bites are rarely hospitalized and die at home. Since 1998 Institut Pasteur in Cambodia (IPC), Phnom Penh has been the only source of free post-exposure prophylaxis (PEP) and *post-mortem* diagnosis.

**Methods:**

The 1998–2007 data compiled by IPC was analyzed to describe all treated patients for PEP, results of human testing and confirmed rabies cases, and results of animal testing. From dog bites' characteristics, we defined a suspected rabid dog bite injury (SRDBI) in humans as a bite that was unprovoked, from a dog that died spontaneously, or from a dog that was reported sick. We applied a deterministic probability model to estimate 2007 rabies human mortality nationwide from the estimated incidence of rabid dog bites, the body distribution of bite wounds, and the probability of PEP access.

**Results:**

During 1998–2007, 124,749 patients received PEP at IPC (average 12,470; range 8,907–14,475), and 63 fatal human cases presenting with encephalitis following a dog bite were reported, in which 73% were confirmed positive for rabies by direct immunofluorescence assay or by reverse-transcriptase polymerase chain reaction. During 1998–2007, IPC tested 1,255 animal brain samples; 1,214 (97%) were from dogs including 610 (49%) positive samples. In 2007, 14,475 patients received PEP (100 PEP/100,000 people in Cambodia) including 95% who resided in Phnom Penh (615 PEP/100,000) or five neighboring provinces. The predictive model estimated 810 human rabies deaths would occur in 2007 (95%confidence interval [CI] 394–1,607), an incidence of 5.8/100,000 (95% CI 2.8–11.5).

**Conclusions:**

Access to PEP is only sufficient for Phnom Penh residents. In 2007, the estimated rabies related mortality exceeded that of malaria and that of dengue. A national rabies control program is needed to improve surveillance and access to PEP, and to initiate vaccination campaigns in dogs.

## Author Summary

In Cambodia, rabies still elicits fear in the communities. Since 1998 the Institut Pasteur in Cambodia (IPC), Phnom Penh has been the only source of free post-exposure prophylaxis (PEP) and *post mortem* diagnosis. During 1998–2007, on average ∼12,400 patients received PEP annually at IPC (range 8,907–14,475) and 63 fatal human cases presenting with encephalitis following a dog bite were reported including 73% who tested positive by fluorescent-antibody test on brain samples or/and by reverse-transcriptase polymerase chain reaction on skin, cerebrospinal fluid, or urine. In 2007, 14,475 patients received PEP (100 PEP/100,000 people in Cambodia) including 95% who resided in Phnom Penh city (615 PEP/100,000) or five neighboring provinces. Using a step-by-step probability model, we estimated that 810 human rabies deaths would occur in 2007 (95% confidence interval [CI] 394–1,607); an incidence of 5.8/100,000 (95%CI 2.8–11.5). As a result, despite high attendance at the IPC's PEP center most Cambodians living in peripheral provinces in Cambodia may not have adequate access to PEP. Finally, the model generated one of the highest incidences of rabies worldwide. A national rabies control program is needed to improve surveillance and access to PEP, and to initiate vaccination campaigns in dogs.

## Introduction

Rabies is a viral zoonotic infection of the nervous central system caused by a lyssavirus and is fatal without proper post exposure treatment [1]. Despite the existence of an effective vaccine, rabies remains a public health problem worldwide, particularly in developing countries where dogs continue to serve as the main reservoir of disease transmission to humans [2],[3]. Globally, animal bite injuries lead to >10 million post exposure treatments per year and an estimated 55,000 people die of rabies each year. However, the number of deaths caused by rabies is considered largely underestimated [4]. Cambodia (estimated 2007 population 14.4 million) is a canine rabies endemic country where data are lacking to properly quantify the burden of the disease in animals and humans [5]. Since 1998 the Institut Pasteur in Cambodia (IPC) in the capital city of Phnom Penh (estimated 2007 population 1.4 million) has been the only source of free post exposure prophylaxis (PEP) and for human and animal rabies laboratory diagnosis. Unfortunately IPC does not have the capacity to take care of patients who developed rabies like symptoms. They are referred to the Calmette hospital which is situated next door. A joint collaboration between the two institutions has led to systematic reports of suspected rabid patients and the possibility for Calmette hospital's clinicians to send samples for laboratory diagnosis of rabies. In absence of national rabies control program and as surveillance of rabies related encephalitis was only phased in throughout the country in 2006–2007, IPC has been the solely reliable source of information on human cases collected in Cambodia. We report the results of retrospective analysis of data from IPC's routine activities during 1998–2007 on (i) all post exposure treated patients, (ii) human rabies cases reported by Calmette hospital and (iii) human and animal specimens testing to describe the epidemiological situation of rabies in Cambodia. Information on dog abundance and rabies vaccination coverage were estimated from several surveys conducted in rural areas in 2005–2007. Finally, we used the 2007 IPC data to estimate the incidence of rabies related human deaths in Cambodia from passively reported dog bite injuries statistics.

## Methods

### Data collection

#### IPC's post exposure treatment center activities

Each patient with animal bites, licking or scratches that presented at IPC from 1998 to 2007 was medically examined and received wound management as required, and a purified Vero cell rabies vaccine injection series according to the intradermal Thai Red Cross regimen and tetanus vaccine injections when appropriate for free [6],[7]. Equine rabies immunoglobulin is recommended for patients with WHO category III exposures; however, due to budget constraints, rabies immunoglobulin is only offered for free to children with wounds on their upper arms and faces. In addition, only patients who found out that the biting animal died, disappeared or was killed within 10 days of the bite were encouraged to come back at D28 for the fourth injection. Since 1998, medical records of all these patients have been set up electronically using Epiinfo version 6.4 d. Details on bite sites/multiple bites/broken skin and biting circumstances either provoked or unprovoked, and the animal's outcomes (i.e. spontaneous death, slaughtered, survived, unknown) were collected alongside demographic information, biting animal's characteristics, time interval between consults and biting events. Dates of each follow-up visit of post exposure vaccination were also recorded. Confidentiality of patients has been safeguarded on a password-secured computerized database only accessible by the IPC rabies clinic's medical staff. For the purpose of the present study, we anonymized medical records by removing patient's identifiers (name and date of birth) and compiled data from 1998–2007 to allow retrospective analysis.

#### Reported suspected patients and patients with confirmed rabies

Since 1998, IPC has kept records of clinically diagnosed rabid patients who presented at IPC or the Calmette hospital, a teaching hospital situated next to IPC. Because of proximity, fresh brain specimens of patients who died could be collected and tested using direct immunofluorescence test for the detection of rabies nucleocapsid antigen [8] and/or by reverse-transcriptase polymerase chain reaction on skin, brain, saliva, urine or cerebrospinal fluid at IPC [9]. We analyzed IPC records which included demographic information, clinical symptoms, body locations of injuries, species of biting animals, geographic origin and reported time interval between bites and onset of signs. Brain biopsies are done routinely at the request of the Calmette hospital's clinicians for confirmation. However the National Ethics Committee and Internal Review Board's approvals were obtained for other testing and presented elsewhere [9].

#### Laboratory diagnosis in animals

Since 1998, IPC has tested brains specimens from dogs and other animals suspected of rabies using direct immunofluorescence assay initially developed for human diagnosis purposes and described elsewhere [8]. No system was in place to actively collect animal specimens. People became progressively aware that animals that appeared sick or/and had bitten can be brought to IPC for free testing for rabies. We have recorded through a standardized form age, sex, species, geographic origins of the suspected animals and dog ownership. Since 2007 additional dog characteristics data have been collected including causes of animal death (spontaneous or killed), provoked or unprovoked biting, altered behavior, and presence of sickness.

#### Dog population estimates in rural areas

The National Veterinary Services do not have a rabies control program and anti-rabies vaccines are delivered. As a consequence, there are no statistics on dog population and the number of vaccinated dogs. We calculated the dog population by estimating dog ∶ human ratios from households surveyed in 151 villages across the country between 2005–2007. A total of 148 different villages were visited as part of four surveys that we conducted on knowledge – attitudes – practices among rural Cambodians regarding H5N1 avian influenza in Kampong Cham, Prey Veng, Svay Rieng, Takeo, Pursat and Banteay Mean Chey provinces using a two-stage household based cluster survey [10],[11]. Three other villages were surveyed as part of an immediate investigation in response to notification of confirmed cases of avian influenza (H5N1) virus infection in Kampot, and Prey Veng provinces [12],[13]. All households located within one- kilometer radius from the H5N1 human cases' households were visited. Among other information in the avian influenza questionnaire by household we included two questions regarding the number of household members and the number of rabies vaccinated and unvaccinated dogs per household. The surveys were approved by the national ethics committee [10]–[13].

### Data analyses

The 1998–2007 data compiled by IPC were analyzed to describe all treated patients, confirmed human rabies cases and laboratory results of human and animal testing. Population estimates were obtained from the 1998 national census accounting for a 1.81% annual population growth [5]. The 2007 number of human deaths caused by rabies *[N]* was calculated as the product of the incidence of suspected rabid dog bite injuries (SRDBI) *[I]*, the population at risk *[Pop.]* and the probability of death following a bite from a SRDBI *[P_death_]*:
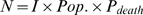
Relying on data collected at the PEP center, we defined a SRDBI as unprovoked biting or from a dog that died spontaneously or from a biting dog that was reported sick. To estimate *I*, the annual incidence of SRDBI for the country, we calculated the SRDBI incidence for Phnom Penh and extrapolated to the national level assuming that the IPC PEP was able to draw all suspected rabid dog bite injuries that occurred in Phnom Penh. We defined “*Pop.*”- human population at risk for rabies as a population in which density of dogs was beyond the density threshold to sustain rabies transmission in dogs (9 per km^2^ by Knobel *et al*) (4).

**Figure 1:**
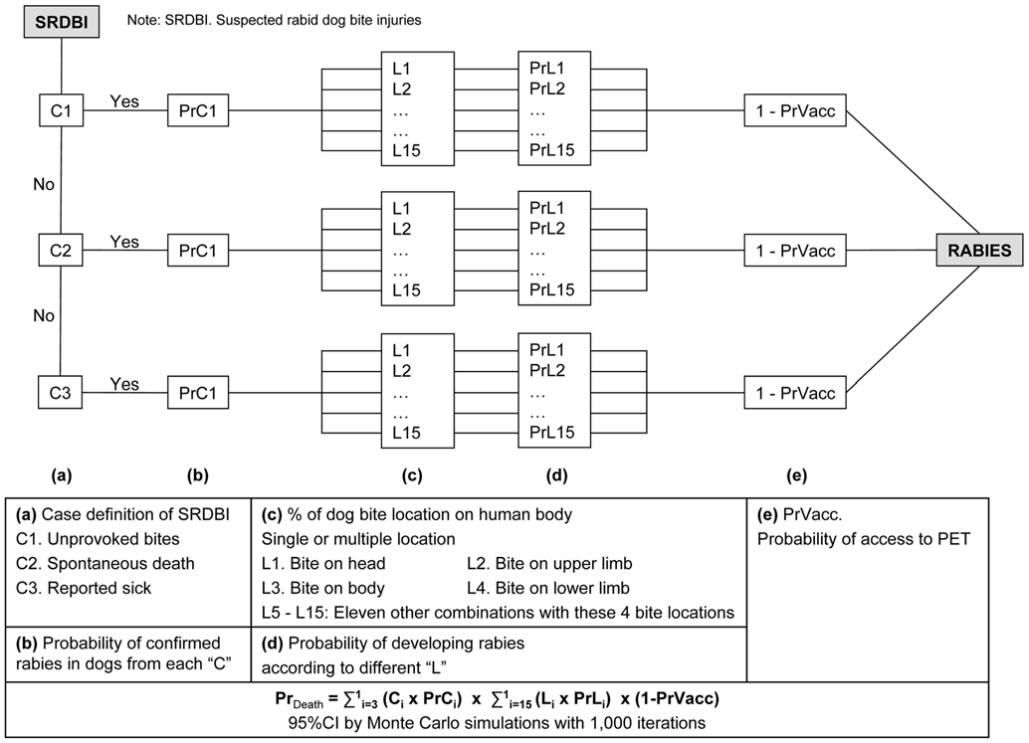
Probability tree for estimating the probability of death following a suspected rabid dog bite injury.

Density of dogs was calculated from the estimated dog∶ human ratio and human population density which derived from the Cambodian National Bureau of Statistics [5]. Finally we adapted the step by step probability model as described by Cleveland *et al* [14] to estimate *P _death_*, the probability of dying following a SRDBI (Figure 1). This model was based on five input parameters which were determined from Cambodian data: (i) proportion of signs that composed SRDBI; (ii) probability of confirmed rabies among suspected rabid dog, which is obtained from laboratory data by comparing positive brain dog specimens with negative ones and calculating the predictive positive values of each of the SRDBI signs when reported alone; (iii) distribution of single and multiple dog bite injuries on human body accounting for single and multiple locations; (iv) probability of developing rabies by body location of bite wounds - we used existing data on estimated likelihood of a person to develop rabies by body location of the bites accounting for rabies probabilities for multiple bite locations; and finally (v) the probability of PEP access which stem from the annual number of PEP center attendees over the population at risk, assuming that PEP was only provided by IPC. We believed the number of people who could afford vaccines provided by the private sector was negligible (Figure 1).

We estimated the confidence limits for the total number of deaths attributable to rabies by bundling probability distributions of input parameters and running Monte Carlo simulations using STATA software version 9.0 (StataCorp, College Station, TX, USA) for 1,000 iterations. The value of each input parameter were chosen at random from within a defined probability distribution and the simulation program produced a probability-based distribution of the net result, which were used to report statistics such as mean and the 2.5th and 97.5th percentiles for 95% confidence intervals (95% CI). Proportions, means, medians, ratios, interquartile ranges (IQR), non-parametric and chi2 tests, p values and 95% CI were calculated using STATA.

## Results

**Figure 2:**
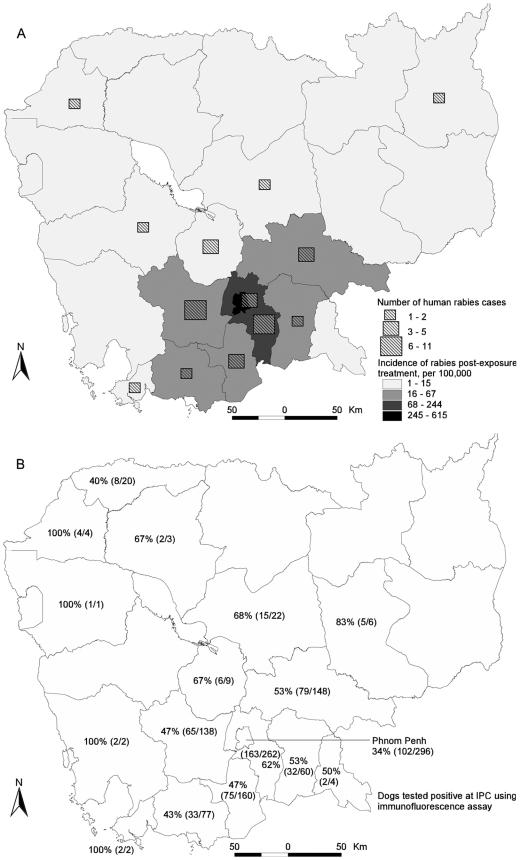
Geographic distribution of rabies in dogs and humans, and incidence of post-exposure treatment, Cambodia, 1998–2007.

During 1998–2007, 124,749 patients attended the IPC's PEP clinic because of an animal bite injury (99.1%) or animal's licking (0.2%) and/or scratches (0.7%). Of the patients with bite injuries, 9.0% presented with deep wounds and 49.0% with multiple bites. The number of patients steadily increased during this period from 8,485 to 14,475 in 1998 and 2007 respectively. In 2007, the median age of the patients was 16 years (range 1–92) with 51.8% males. The overall Cambodia PEP rate was 101/100,000 this year and varied by province from 0.9 to 615/100,000 with a median rate of 7.2/100,000. The highest rates were observed in Phnom Penh and the five neighboring provinces (Figure 2.A) which accounted for 59.5% and 36.7% of the total number of cases, respectively. Among the attendees in 2007, 95.7% reported dog bite injuries of which 10,437 (75.3%) met the case definition of SRDBI. Of these SRDBI, 95% of them consulted the IPC clinic within 3 days of a bite injury (median duration 1 day; range 0–112 days). Adherence to prophylaxis is high; 95% of patients come back at D7 of the treatment – particularly among Phnom Penh residents compared with patients living beyond Phnom Penh (95.9% versus 94.4%, p<0.001). Only 8.7% showed up at D28 for the fourth injection as these patients were those for whom the biting dog was killed, missing or tested positive at IPC.

During 1998–2007, among 63 patients who were admitted to the Calmette hospital with encephalitis following a dog bite (mean 7 per year, range 0–18 patients), none had a reported history of vaccination against rabies. Of these, 60 had their biological samples collected and tested at IPC; 44 (73%) tested positive for rabies, either by reverse-transcriptase polymerase chain reaction (57%, 20/35) of skin, brain, saliva, urine or cerebrospinal fluid, and/or by direct immunofluorescence assay on brain samples (96%, 24/25).

Among the 44 positive cases, 37% were 15 years old or younger (median age 26 years; range, 6–67) and 28 (63%) were males. Thirty-eight (87%) patients resided within 200 km from IPC (Figure 2.A) including 13 (20.6%) and 4 (9%) in Kandal province and Phnom Penh respectively. The last case who reported to have been bitten by a dog in Phnom Penh city dated from 2006. The reported median incubation period calculated from 35 patients was 60 days (IQR range 30 to 100 days). Symptoms that were observed among rabid patients included acute behavior changes or/and hyperactivity/excitability (98%), hydrophobia (86%) and aerophobia (55%). Of the 15 patients presenting with encephalitis but negative for rabies, these symptoms were seen in 87%, 53% and 47%, respectively. Patients were admitted to the hospital approximately 2 days on average (range 1–8) of the onset of symptoms.

From 1998 through 2007, the IPC laboratories received 1,255 animal heads; dog heads made 96.7% of the animals of which 49.2% tested positive for rabies antigens in brain tissue. Other animals included cats (17.6% positivity), bovines (90.9%) and monkeys (12.5%). Except for bovines, all other heads stem from biting animals. Of the 596 laboratory confirmed rabid dogs, 67% were male and the median age was 18 months ranging from 2–120 months. Rabid dogs were confirmed in 17 Cambodia provinces; however, 95% of them were located within 200 km from Phnom Penh including 44.5% which lived in Phnom Penh or Kandal provinces (Figure 2.B). In 2007, 109 (51%) of 214 dogs tested positive and originated from 14 provinces. When comparing the characteristics of the rabid dogs with positive brain specimens with the negative specimens in 2007, the positive predictive value was highest for reported sick biting dogs (93% , 95%CI 66%–100%), followed by dogs that died spontaneously 17% (95%CI 1%–64%) and for biting from an unprovoked aggression (12%, 95%CI 6–22%) (Table 1).

**Table 1:** Positive predictive values of suspected rabid dog characteristics when reported alone - dogs from 14 provinces in Cambodia, 2007.

Clinical Characteristics in Dogs		Virology Testing		Positive Predictive Values
		Negative	Positive	
Reported sick alone (n = 38)	Yes	1	13	93%
	No	24	0	0
Spontaneous death alone (n = 30)	Yes	5	1	17%
	No	24	0	0
Unprovoked bites alone (n = 105)	Yes	71	10	12%
	No	24	0	0

Of the 1,538 households of 151 villages in seven rural provinces that were surveyed during 2005–2007, 75% of the households owned at least one dog. A total of 2,670 owned dogs were recorded for 8,269 individuals surveyed yielding a ratio of 1 dog to 3.1 humans (95%CI 1∶3.0–1∶3.2). Only 17 (1.4%) dogs were reported to have been vaccinated against rabies. No significant differences in the ratios were observed between the seven provinces.

In 2007, of the 8,606 patients who attended the IPC's PEP rabies clinic and resided in Phnom Penh, 5,398 (62.7%) were bitten by a dog and had an injury that met the case definition of a SRDBI. These reported SRDBI included inflicted by sick dogs (0.4%), by dogs that died spontaneously (1.0%) or by unprovoked dog aggression (99.3%). Of these, most bites were located on lower limbs (59.3%) followed by upper limbs (22.8%), trunk (10%) and the head 5.7%; multiple biting (>1 wound) accounted for 93.2%. Assuming that the IPC clinic was able to capture a large majority of SRDBI that occurred in Phnom Penh, the Phnom Penh incidence of SRDBI would be at least 386 per 100,000 (95% CI: 374–394/100,000). When extrapolating this incidence of Phnom Penh to the entire country, 53,732 injuries at risk for rabies (SRDBI) would have occurred in Cambodia. As a consequence, the model yielded a probability of rabies-related death from a suspected rabid dog bite of 1.51% (95%CI: 0.76–2.93%) which resulted in 810 (95%CI: 394–1,607) deaths and a rabies incidence rate at 5.8/100,000 (95%CI: 2.8–11.5/100,000) for 2007 in Cambodia (Table 2).

**Table 2:** Model parameters for estimating the probability of human deaths caused by rabies virus infection, Cambodia, 2007.

Estimation parameters, year 2007	Mean	Minimum	Maximum
**Estimated Incidence of rabies death in Cambodia, per 100,000**	**5.8**	**2.8**	**11.5**
**Number of rabies death in Cambodia ** ***(N)***	**810**	**394**	**1,607**
**Incidence of SRDBI in Cambodia, per 100,000 ** ***(I)***	**386**	**374**	**394**
Number of SRDBI in Cambodia	53,728	52,062	54,846
**Incidence de SRDBI in Phnom Penh, per 100,000**	**386**	**374**	**394**
Number of SRDBI in Phnom Penh	5,398		
**Number of dog bite injuries in Cambodia**	**82,268**	**80,459**	**84,078**
Incidence of dog bite injuries in Phnom Penh, per 100,000	591	578	604
Number of dog bite injuries in Phnom Penh	8,263		
Number of patients receiving PEP at IPC	14,475		
**Probability of death following a SRDBI ** ***(P death) (%)***	**1.51**	**0.76**	**2.93**
**Clinical characteristics of biting dogs (%)**			
Unprovoked bites (C1)	96.26	95.86	96.63
Spontaneous death (C2)	1.47	1.23	1.73
Reported sick (C3)	2.27	1.98	2.59
**Probability of confirmed rabies in dogs from each “C” (%)**			
Unprovoked bites (PrC1)	12.00	6.00	22.00
Spontaneous death (PrC2)	17.00	0.42	64.00
Reported sick (PrC3)	93.00	66.00	100
**Proportion of dog bite location(s) on human body (%)**			
Head [H] (L1)	5.72	5.34	6.12
Upper limb [UL] (L2)	22.83	22.13	23.54
Body [B] (L3)	10.11	9.61	10.62
Lower limb [LL] (L4)	59.28	58.45	60.10
H-UL (L5)	0	0	0
H-B (L6)	0.34	0.25	0.45
H-LL (L7)	0	0	0
UL-B (L8)	0.70	0.57	0.85
UL-LL (L9)	1.03	0.86	1.21
B-LL (L10)	0	0	0
H-UL-B (L11)	0	0	0
H-LL-B (L12)	0	0	0
H-UL-LL (L13)	0	0	0
UL-LL-B (L14)	0	0	0
H-UL-B-LL (L15)	0	0	0
**Probability of developing rabies for different “L” (%)**			
Head [H] (PrL1)	45.00	30.00	60.00
Upper limb [UL] (PrL2)	28.00	15.00	40.00
Body [B] (PrL3)	5.00	0.01	10.00
Lower limb [LL] (PrL4)	5.00	0.01	10.00
H-UL (PrL5)	60.40	40.50	76.00
H-B (PrL6)	47.80	30.00	64.00
H-LL (PrL7)	47.80	30.00	64.00
UL-B (PrL8)	31.60	15.00	46.00
UL-LL (PrL9)	31.60	15.00	46.00
B-LL (PrL10)	9.80	0.02	19.00
H-UL-B (PrL11)	61.10	40.50	73.60
H-LL-B (PrL12)	50.10	30.00	66.40
H-UL-LL (PrL13)	61.10	40.50	73.60
UL-LL-B (PrL14)	34.90	15.00	50.60
H-UL-B-LL (PrL15)	62.80	40.50	74.80
**Probability of access to PEP (Pr Vacc) (%)**	**17.60**	**17.34**	**17.86**
At risk population in Cambodia ***(Pop.)***	13,920,243	13,920,243	13,920,243
Population of Phnom Penh	1,398,555	1,398,555	1,398,555

Note: SRDBI: Suspected rabid dog bite injuries; PEP: Post-exposure treatment; IPC: Institut Pasteur in Cambodia.

## Discussion

This report is the first published national data on rabies in Cambodia and confirms that rabies remains a serious public health hazard where dog bites continue to be the main source of transmission. Clearly rabies transmission has also occurred in Phnom Penh capital city and its vicinity even though the population is more aware of the disease (Cambodia Ministry of Health/UNICEF's unpublished data). Over 95% of the PEP patients come from Phnom Penh and five surrounding provinces. PEP coverage may only be satisfactory for Phnom Penh residents with rates that are similar to that of Vietnam or Thailand whereas access rates to PEP dropped out as distance from IPC increases [15],[16]. It is likely this pattern only reflects a catchment's area of IPC instead of the true geographic distribution of rabies in Cambodia. This high attendance among Phnom Penh population combined with high adherence to treatment showed rabies still elicits fear in Phnom Penh. This may be probably true also for the rest of the country as a local name for rabies known even among children (“mad dog” disease) exists and is a reflection of a long history of the disease among Cambodians. Reasons for low attendance among people residing in remote provinces should be explored. Whether these people were less knowledgeable about a free PEP provided by IPC remained unclear. It is possible that despite free treatment in Phnom Penh, a series of rabies PEP injections with day intervals could deter poor people living in remote and rural areas from back-and-forth travels to IPC. This finding is of utmost importance to the health authorities as to underscore the needs for Cambodia to have more than one post exposure treatment center. Further investigations are needed to identify areas in which a second or a third treatment center would be appropriate.

Our second major finding was a remarkably large population of dogs compared to that of humans; we found 1 dog for approximately every 3 humans, a ratio that was 3–4 times higher than that of neighboring countries. To our knowledge, worldwide this magnitude is second to the highest ratio observed in Sri Lanka with a ratio of 1∶2.4. We estimated that the dog population at 4.4 million considering an 84% dog ownership [17] and 80% of Cambodians living in rural areas [5]. Commonly the dog ∶ human ratio is lower in urban areas [4]. If we assumed this ratio to be similar to that of Thailand (two-fold lower in urban areas compared to rural areas), the total number of dogs in Cambodia could be up to 5 million. The abundance of dogs in Cambodia was not a surprise and likely to be explained by several factors: (i) as a Buddhist country, Cambodians are reluctant to kill or eat dogs; (ii) dogs are popular as they are useful for guarding houses and (iii) birth control in dogs is rarely available, particularly in rural areas. Taken together with a potentially rapid growing dog population and an inexistent canine vaccination against rabies, rabies situation in the country could become alarming. On the other hand, despite this large dog population, a relatively high frequency of dog ownership described in this country could allow a substantial proportion of dogs be accessible for canine parenteral vaccination against rabies and therefore reach the 70% herd immunity to interrupt rabies transmission in dogs [18]–[20].

The model revealed a high incidence of rabies related human deaths (5.8 per 100,000) in Cambodia, which was 15 times higher than that of the official reports. To put it into perspective, rabies caused more deaths than dengue or malaria related deaths in Cambodia (∼100 and ∼400 deaths on average for the past 5 years caused by dengue and malaria respectively). These estimates of dengue and malaria related deaths are produced by the Dengue and Malaria National Control Program from national surveillance systems [21]. This leaves Cambodia among countries with the highest incidence in the region – followed by India with an estimated incidence of 2–3 per 100,000 [22]. This discrepancy in the numbers of rabies related deaths between the official reports and our model was the result of the absence of surveillance and limited diagnostic capacity in Cambodia. Nevertheless, although rabies has been listed as a priority disease under surveillance since 2006, it is not a surprise that rabies related encephalitis would still be underreported because anecdotal reports suggest that poor patients with encephalitis following dog bites are rarely hospitalized and die at home. As a consequence rabies is probably not seen by the authorities as a significant public health problem.

It is important to interpret our results in light of some limitations. The model strongly relied on two major assumptions. First, we reasonably speculate that a large majority of Phnom Penh residents bitten by a dog would attend the PEP center– particularly those who were bitten by suspected rabid dogs because the IPC's PEP center is easily accessible and has become a well-known institution within Phnom Penh and rabies still generates fear in the communities. As a result, it is plausible that our estimate is close to the true incidence of SRDBI in Phnom Penh. The extent to which dog bite injuries were seen and treated by the private sector is difficult to estimate; however the number may be negligible compared to that of IPC PEP center as only a small population could afford cell culture-based vaccines against rabies at a prohibitive price. Second, the incidence of suspected rabid dog bites in Phnom Penh is supposed to be similar to that of the rest of the country – yet Cambodia is mainly rural. However, it is well recognized that incidences in rural areas are commonly higher than that of cities [4],[23]. Therefore we believe the model has predicted a conservative estimate of rabies incidence. Indeed, our two assumptions if inaccurate, only affected the model by underestimating the true burden of rabies in rural Cambodia.

Nevertheless, despite these limitations, we have shown that laboratory data can provide important information on generating predictive positive values of rabies infection for parameters that were accounted for in the model. Countries where such diagnostic capacity exists should be encouraged to collect data regarding dogs or dog bites so that each parameter needed for the decision tree model could be validated locally. Combined with additional epidemiological studies - surveys or sensitive/active surveillance system of encephalitis - to estimate the incidence of a suspected rabid dog bite injury such countries would be able to provide country level estimates needed for advocacy and setting priority in Asia and in Africa [2],[3].

We believe that the present analysis represents an important contribution in bringing rabies to the attention of Cambodia national authorities where rabies is often perceived as a rare disease because of the lack of incidence data. Extrapolating from the model, the current PEP center for the past 10 years may have been prevented ∼1,000 rabies related deaths. However, only one free PEP clinic in Phnom Penh is not sufficient to handle a country in which rabies transmission occurs endemically. Since 1997, IPC has spent approximately US$ 1.2 million on PEP excluding the cost of anti-tetanus vaccines and laboratory diagnostic costs (IPC unpublished data). It is unlikely that this financial burden could eventually be reduced as more and more people could afford to reach the capital city in the future and if little is done to mitigate transmission of rabies virus in a plausibly growing dog population. Therefore, we strongly recommend establishing a comprehensive national rabies control program whose one of the major challenges would be to work across ministries and agencies to ensure continued political commitment and active community participation so that proper WHO recommended rabies vaccines are available and accessible to Cambodians and rabies transmission in the dog population is controlled. Progress on vaccine administration in humans and dogs, recent success in Sri Lanka or many South East Asian countries regarding rabies control program, and mounting evidence of cost effective interventions for rabies elimination should encourage Cambodia to tackle this fatal but preventable disease [24]–[29].
